# Optimal timing of treatment for errors in second language learning – A systematic review of corrective feedback timing

**DOI:** 10.3389/fpsyg.2023.1026174

**Published:** 2023-02-23

**Authors:** Mingfei Xu, Simin Zeng

**Affiliations:** ^1^College of International Education, Minzu University of China, Beijing, China; ^2^School of Humanities and Social Sciences, Harbin Institute of Technology (Shenzhen), Shenzhen, China

**Keywords:** corrective feedback, feedback timing, second language acquisition, interaction, systematic review

## Abstract

Although a large body of research has developed on corrective feedback in second language acquisition (SLA) in the past 30 years, there are few empirical studies examining the relationship between feedback timing and SLA. To begin to address this gap, this study reviews the existing research on the impact of corrective feedback timing on SLA. It aims to investigate the possible influential factors that might have led to inconsistent research findings and theoretical explanations. The review was conducted according to PRISMA-statement through searches in peer-reviewed electronic databases including Social Sciences Citation Index (SSCI), Scopus, EBSCO, which includes ERIC and the British Education Index and gray literature (doctoral dissertations in ProQuest). Twenty studies conducted and published between 2006 and 2021 were finally analyzed to reveal the current trends. The results of this review indicate that there is no definite answer to the question of when errors in L2 should be treated. The difficulty of drawing a conclusive finding can be attributed to the communicative modality examined and variations in research design, including the explicitness of feedback and various ways of measuring feedback timing. No certain theoretical framework has been applied to guide these studies and they have applied different theoretical explanations to interpret the inconsistent results. The review highlights the need to continue to investigate the effectiveness of corrective feedback under different timing conditions. In addition, it discusses some research gaps that should be addressed in future studies and suggests future research directions in the area of feedback timing.

## 1. Introduction

Corrective feedback (*CF*) ‘refers to any signal that a learner’s utterance may be erroneous in some way’ (Nassaji and Kartchava, 2021, p. 1). It is also known as negative evidence in SLA literature (Gass, 2003). Following decades of *CF* research, there has been a consensus that *CF* is beneficial to L2 learning. The most widely investigated topics include what types of *CF* are more effective (Ammar and Spada, 2006; Sheen, 2010), what factors influence the effectiveness of *CF* (Sheen, 2008; Goo, 2012; Li, 2014), the positive effects of *CF* on cognitive processes such as attention (Moret-Tatay et al., 2016, 2022) and teachers’ and learners’ beliefs about *CF* (Li, 2017), etc. Despite the abundance of the acceptance of feedback in L2 learning, the timing of correction is still a point of contention among researchers. *CF* timing refers to when errors in L2 are treated (Quinn, 2021). *CF* provided directly after an error is made is immediate *CF*, while *CF* provided at a later time is delayed *CF*. Dated back to the first synthesis of corrective feedback research, the second fundamental question raised by Hendrickson (1978) is “when should learners’ errors be addressed?” (p.389). However, it was not until recent years did researchers start to investigate *CF* timing as an independent variable.

As far as research is concerned, many studies have examined the effect of feedback timing in the field of cognitive psychology (Kulik and Kulik, 1988; Butler et al., 2007; Metcalfe et al., 2009; see Nakata, 2014 for a review). It was not until recent years did researchers begin to directly compare the efficacy of immediate and delayed feedback on L2 learning. Among these few studies, they have not shown a clear difference between the two timings of feedback. The investigation of *CF* timing in SLA is recent popularity probably because it is widely accepted that immediate *CF* is a common practice in oral *CF* in L2 learning and teaching. The motivation to investigate how delayed *CF* facilitates L2 learning and the comparative effectiveness with immediate *CF* is comparatively less strong (Quinn and Nakata, 2017). Also, there is a lack of widely accepted theoretical explanations about how L2 learners could benefit from delated *CF*. However, from a pedagogical perspective, teachers and practitioners care much about when feedback should be provided. On the whole, corrective feedback in L2 has been widely researched but the key question of when to provide *CF* remains unanswered. In this case, a general overall picture of the evidence in a *CF* timing is needed to direct future research efforts, and more importantly an accurate picture of existent research, and methodological issues are required. A systematic review of *CF* timing could investigate international empirical research evidence to inform future practice and research.

## 2. Theoretical explanations

The timing of corrective feedback refers to ‘the juncture in the instructional sequence when learners’ errors are addressed’ (Quinn and Nakata, 2017. p.35). The optimal time to provide *CF* has attracted theoretical debates, but most SLA theories do not make overt claims about the ideal time to provide *CF*. The effectiveness of immediate feedback can firstly be explained by behaviorism, which treats feedback as a device that corrects errors and reinforces correct behaviors (Skinner, 1953). Learning is thus viewed as habit formation and errors should be immediately corrected, otherwise, they could turn into bad habits. The utility of immediate correction is also advocated by the skill acquisition theory (SAT; Anderson, 1983; DeKeyser, 2015). According to SAT, explicit knowledge becomes implicit knowledge through practice and researchers advocate the use of prompts. Prompts, according to Ranta and Lyster (2007) are more likely to encourage learners to retrieve a learned grammar rule and reattempt to produce the language more accurately with that rule in mind and thus can facilitate the proceduralization of previous knowledge. Therefore, when aiming to facilitate the proceduralization of the knowledge, *CF* must be provided during communicative interaction, and during not after communicative tasks. As such, it seems that SAT explains the effectiveness of immediate *CF*. The third theoretical support for immediate *CF* is the immediate cognitive comparison. Certain types of *CF* such as recasts or reformulations provide learners with the opportunities to have an immediate cognitive comparison between their errors and the accurate models (Doughty, 2001). Doughty (2001) further argues that the effective comparison should occur within the “cognitive window of opportunity” (p. 257), which is about 40 s later as long as the learner could hold a representation of the propositional message. As this theoretical support highlights the importance of immediate *CF* after errors during communication, it also supports the beneficial role of immediate *CF* in SLA. The last theory that can be drawn on to support immediate *CF* is Sociocultural theory (SCT; Aljaafreh and Lantolf, 1994). According to SCT, *CF* should be provided in a tailored, graduated and contingent way, which can only be provided in the learners’ zone of proximal development (ZPD). In this case, *CF* should be dynamically provided as a tailored response to learners’ changing needs.

On the other hand, the theoretical arguments for the utility of delayed *CF* are comparatively less. A cognitively oriented model related to *CF* timing is Transfer Appropriate Processing (TAP; Morris et al., 1977; Lightbown, 2008), which indicates that immediate and delayed *CF* may result in the development of different types of L2 knowledge. Based on this theory, the context where learned knowledge is retrieved and applied must be consistent with the context where the knowledge is acquired (Li, 2020). As a result, providing immediate *CF* that is integrated into synchronous oral or written communication may lead to an increase in procedural grammar knowledge while providing delayed *CF* following communicative discourse may lead to an increase in explicit knowledge. Another theoretical perspective that supports the advantage of delayed feedback is Spacing Theory, which emphasizes that maximal learning occurs through ‘repeated presentations that are spaced (distributed) as opposed to massed’ (Butler et al., 2007, p.274). In this case, massed instruction provided in immediate feedback imposes more cognitive burden than spaced instruction in delayed or separating feedback. The reactivation and reconsolidation theory (Nader and Einarsson, 2010) in cognitive psychology also predicts the advantage of delayed over immediate feedback. It argues that when a learner is reminded of a previously learned pattern, a long-term mental representation of it is activated. The memories become labile and susceptible to influence and learners’ labile state allows for reconsolidation, which is retrieval-induced and occurs in both declarative and procedural memory systems. Quinn (2014) argues that although both immediate and delayed *CF* can stimulate the retrieval and reconsolidation of linguistic forms, delayed *CF* is more effective since more time can be allocated for both retrieval and reconsolidation to take place.

In summary, despite the differences in the theoretical positions supporting immediate or delayed *CF*, they provide possible explanations as to why immediate or delayed *CF* is comparatively more effective. However, deciding when to provide *CF* is an empirical question which has received little investigation.

## 3. Methodology

The principal aim of this review was to analyze and synthesize empirical literature on feedback timing in SLA. When there are too few studies to yield data in an immature research field, it has been argued that a literature review may be less valuable. A systematic review rather than a scoping review was conducted. Scoping reviews may be conducted as a first step of systematic reviews (Petticrew and Roberts, 2006) to “identify knowledge gaps, scope a body of literature, clarify concepts or to investigate research conduct” (Munn et al., 2018, p.1). However, this study takes a further step to investigate conflicting results of each study and identify trends in the current evidence to provide suggestions for future studies. In this situation, a systematic review can highlight the absence of data, and point to the fact that any understanding is based on limited empirical underpinnings. Thus, this study conducts a systematic review, which ‘adheres closely to a set of scientific methods that explicitly aim to limit systematic error (bias), mainly by attempting to identify, appraise and synthesize all relevant studies’ (Petticrew and Roberts, 2006, p. 9). The design and reporting of the results of this systematic review were conducted according to the PRISMA statement (Moher et al., 2009, 2015). The research protocol used included designing research questions, creating the search strategy to collect studies; a selection of scientific databases, criteria for the inclusion and exclusion for studies; and an approach for selection, extraction, and analysis.

### 3.1. Research questions

This systematic review aimed to answer the following research questions:

1. What are the main influential factors that affected the comparative effectiveness of immediate and delayed feedback in the literature?
2. What theoretical explanations have been applied to explain the results?

### 3.2. Research strategies

Searching for the appropriate studies is the most important step in conducting a systematic review. Both published and unpublished studies including PhD dissertations were included to help alleviate publication bias (Rosenthal, 1979). The research of the international literature was conducted in the following peer-reviewed electronic databases including Social Sciences Citation Index (SSCI), Scopus, EBSCO, which includes ERIC and the British Education Index Index and gray literature (doctoral dissertations in ProQuest). The World Wide Web (like Google academic search) was also used as an additional resource to search for relevant empirical studies on feedback timing in SLA. We also searched the reference lists of records selected and forward citations to identify all relevant publications.

These databases include leading publications on SLA and thus, are considered to be reliable for searching the latest research in this field. The literature search was conducted from October 5, December 2021 to August 6, 2022. We used the following key words: (feedback OR corrective feedback OR *CF*) AND (feedback timing OR immediate feedback OR delayed feedback OR synchronous computer-mediated communication OR SCMC OR asynchronous computer-mediated communication OR ACMC) AND (L2 learning OR second language learning OR second language acquisition OR SLA OR language), etc. The generality of the keywords was purposely selected to include all the categories of feedback and feedback timing identified in the previous literature.

There were no time limitations among the publications of the articles, books, book chapters, or doctoral dissertations. The search covered titles, authors, abstracts, and keywords to minimize irrelevant articles. The selection strictly followed the inclusion and exclusion criteria. The two researchers searched the databases independently. They screened the abstracts to determine which studies fitted the inclusion criteria. Then these studies were examined more closely with a reading of the full papers in order to confirm their eligibility for the subsequent analysis. All these studies were examined by the two researchers to ensure consensus in their adequacy. Employing Cohen’s Kappa, inter-rater reliability was estimated as 0.96 indicating a high degree of consensus between two researchers. The few disagreements were fixed through a consensus between researchers referring to the source data.

### 3.3. Inclusion and exclusion criteria for studies

To be included in this systematic review, this study followed a set of predetermined criteria for the inclusion and exclusion of articles. Studies should follow the following combined criteria:

1. The study had to have been published in English.
2. Only intervention or experimental studies were included.
3. The study must have compared the effects of immediate and delayed feedback in L2 learning.
4. The effects of feedback timing should have been measured.
5. The study should have been published in a peer-reviewed scientific journal or be a book chapter or an unpublished doctoral dissertation.

The following exclusion criteria were applied during the selection process:

1. The study was written in a language other than English.
2. The study reported either only the effectiveness of immediate feedback or only delayed feedback without comparing them.
3. The study was published in a journal that was not peer-reviewed or was an MA dissertation/thesis, systematic review, meta-analysis or commentary, conference proceedings, or working paper.

**Figure 1:**
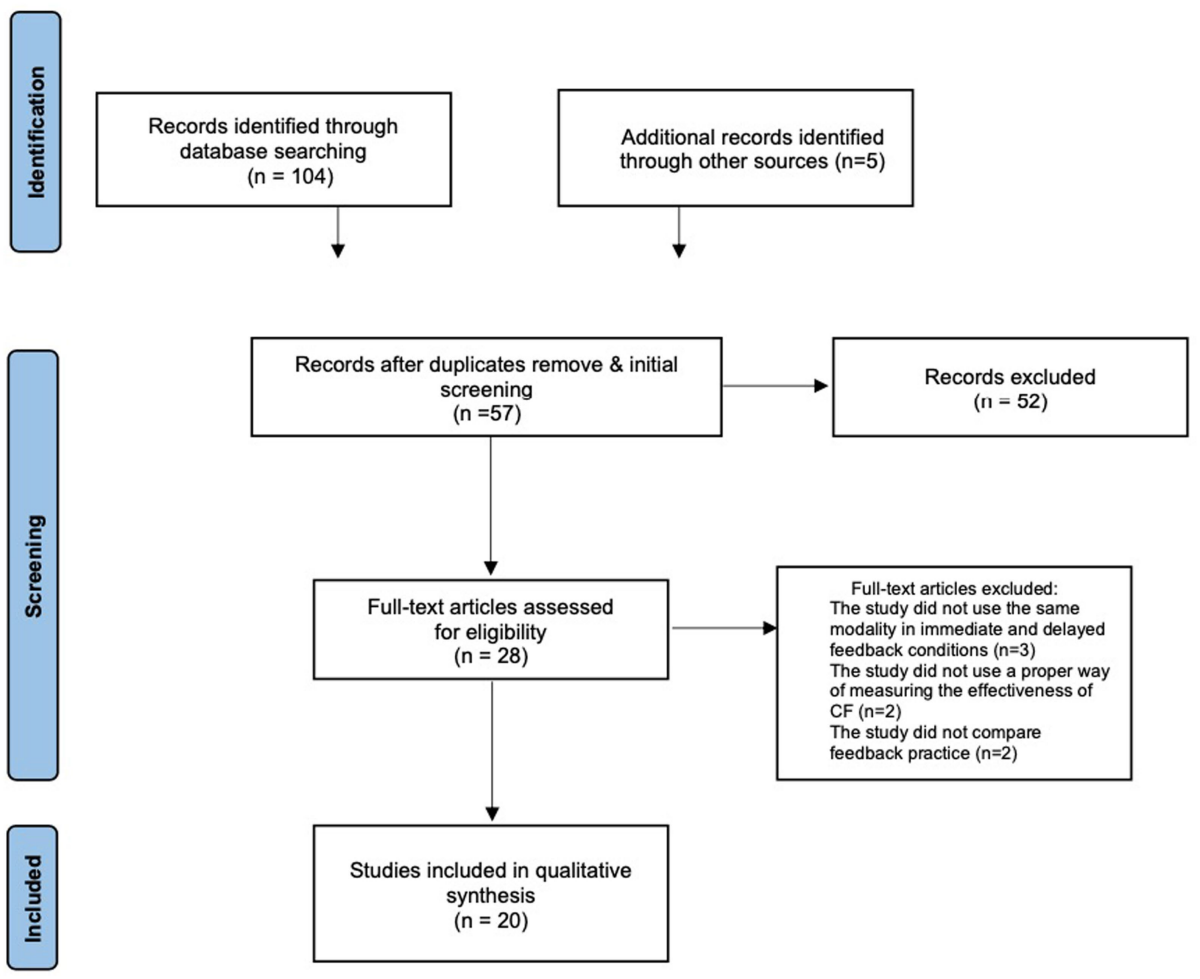
PRISMA flow chart of study selection.

This first search identified a total of 109 potentially eligible articles. After removing duplicates and initial screening of the titles, 57 studies remained in the pool. The two authors then analyzed the titles and abstracts separately. Then they discussed the results of the reviewed studies and excluded 29 studies, leading to 28 studies to assess for eligibility based on full-text analysis. The first and second author read the 28 full-text studies and independently analyzed whether they met the inclusion/exclusion criteria. After independent analysis, the authors discussed the results to determine which studies should be included in the synthesis. The full-text analysis led to the elimination of 8 studies: 3 because of applying different modalities in immediate and delayed *CF* conditions, 2 because of using improper ways of measuring the effectiveness of *CF*, and 2 because of not comparing the practice of feedback (see Figure 1 for the flow chart). 20 studies were included in the systematic review (see Table 1 for details). According to Petticrew and Roberts (2006) and Gough et al. (2012), the number of studies included in a systematic review mainly depends on the research topic and all available studies related to that question. Although the number of included studies seems to be small, Gough et al. (2012) argued that to find the few that are on topic, a lot of irrelevant studies have to be sifted through. Given feedback research almost invariably examines immediate feedback, feedback timing is a narrow scope and a new territory in feedback research and in SLA research in general. In this case, the amount of supportive evidence available is limited (Table 2).

**Table 1:** Studies included in the systematic review.

Author and year	Sample	L1 → L2	Communication mode	Feedback type	Feedback timing	Key findings
Arroyo and Yilmaz (2017)	45 adults	English→ Spanish	Text-based CMC	Reformulations	Immediate *CF*: immediately following the non-target like productions during task; delayed *CF*: at the end of the task	Immediate feedback was more effective than delayed feedback
Arroyo and Yilmaz (2018)	45 adults	English→ Spanish	Text-based CMC	Reformulations	Immediate *CF*: immediately following the non-target like productions during task; delayed *CF*: at the end of the task	Immediate feedback was more effective than delayed feedback in OPT
Canals et al. (2021)	52 adults	Spanish→ English	Video-based CMC	Immediate *CF*: explicit *CF*; delayed *CF*: edited video recording of the interaction	Immediate *CF*: during the task delayed *CF*: 24 h later	No significant differences between immediate and delayed *CF*
Fu and Li (2019)	106 teenagers	Chinese → English	Face-to-face oral	Corrective recasts	Immediate *CF*: session 1 delayed *CF*: session 3	Immediate *CF* was more facilitative than delayed *CF*
Fu and Li (2020)	145 teenagers	Chinese → English	Face-to-face oral	A prompt followed by a recast	Immediate *CF*: in the first session delayed *CF*: in the final session	Immediate *CF* was more facilitative than delayed *CF*
Guo (2018)	60 undergraduates (17–21 years old)	? → English	Face-to-face oral	Proactive approach of peer-review and whole-class discussion	Immediate *CF*: after the initial test delayed *CF*: at the beginning of the next class session	Delayed *CF* was more facilitative than immediate *CF*
Henderson (2021)	30 adults	English→ Spanish	Text-based CMC	Error repetition + recast	Immediate *CF*: immediate after an error delayed *CF*: when the task was finished	No significant differences between immediate and delayed *CF*
Henshaw (2011)	102 adults	English→ Spanish	CALL	Explicit feedback+ one-sentence metalinguistic explanation	Immediate *CF*: immediate after each response submission; first delayed *CF*: immediately after all 40 responses; second delayed *CF*: 24 h after all responses	No significant differences between immediate and delayed *CF*
Lavolette (2015)	118 adults	Chinese+ Arabic → English	CALL	With or without metalinguistic information	Item-by-item or end-of-test	No significant differences between immediate and delayed *CF*
Lavolette et al. (2015)	32 adults	Majority Chinese→ English	CALL	Copying and pasting their work from TextEdit into Criterion, then clicking a button to submit it	Immediate *CF*: immediate after writing; delayed *CF*: 1 to 3 weeks after writing	No significant differences between immediate and delayed *CF*
Li et al. (2016a)	120 teenagers	Chinese → English	Face-to-face oral	Prompts + recasts	Immediate *CF*: immediate after an error delayed *CF*: after the second oral task	Immediate *CF* was more facilitative than delayed *CF* in lower-level learners
Li et al. (2016b)	120 teenagers	Chinese → English	Face-to-face oral	Prompts + recasts	Immediate *CF*: immediate after an error delayed *CF*: after the second oral task	No difference in EIT scores, immediate *CF* was found more effective than delayed feedback in GJT
Nakata (2014)	98 teenagers	Japanese→ English	CALL	Immediate *CF*: The target English word, Japanese translation, and learners’ response were given in the feedback window. Feedback also indicated whether the response was correct, partially correct, or incorrect. delayed *CF*: Feedback for all eight delayed feedback items was presented one at a time	Immediate *CF*: immediate after each response; delayed *CF*: not given until the end of each retrieval phase	No significant differences between immediate and delayed *CF*
Quinn (2021)	90 adults	Various→ English	Face-to-face oral	Prompt + reformulation	Immediate *CF*: Immediate after an error; delayed *CF*: at the end of the task	No significant differences between immediate and delayed *CF*
Rahimi and Vahid Dastjerdi (2012)	20 teenagers	Iranian→ English	Face-to-face oral	Not mentioned	Immediate *CF*: during the speech delayed: after finishing the speech	Delayed *CF* has positive effect on fluency and accuracy but not on complexity
Shang (2017)	44 adults	Chinese → English	Text-based SCMC+ CALL	Immediate *CF*: CALL delayed *CF*: CMC text-based from peers	Immediate *CF*: immediate after the writing delayed *CF*: at the convenience of the learners	ACF was more effective than SCF
Shintani (2015)	2 adults	Japanese → English	Text-based CMC	Focused direct feedback	Synchronous *CF*: immediate after an error; Asynchronous *CF*: after the writing task	SCF was more effective than ACF
Shintani and Aubrey (2016)	68 adults	Japanese → English	Text-based CMC	Focused direct feedback	Synchronous *CF*: immediate after an error; Asynchronous *CF*: after the writing task	SCF was more effective than ACF
Varnosfadrani (2006)	28 immediate level adults	Iranian→ English	Face-to-face oral	Immediate *CF*: explicit feedback delayed *CF*: a reminder of the error + explicit *CF*	Immediate *CF*: during the interview delayed feedback: after the interview	No significant differences between immediate and delayed *CF*
Yilmaz and Sağdıç (2019)	43 adults	English→ Spanish	Text-based CMC	Reformulations	Immediate *CF*: immediate after the errors; delayed *CF*: at the end of the task	Immediate *CF* was more facilitative than delayed *CF* in OPT

**Table 2:** Summary of the main results.

Research questions	Summary of main results
RQ 1: What are the main influential factors that affected the comparative effectiveness of immediate and delayed feedback in the literature	**1.** Communicative modality Face-to face communication—mixed findings Text-based communication—immediate feedback is argued to be more effective Computerized feedback in the CALL environment–no significant difference Video-based communication–no significant difference
	**2.** The explicitness of the feedback Explicit feedback–most studies found no significant difference Implicit feedback–immediate feedback is argued to be more effective Hybrid feedback–immediate feedback is argued to be more effective
	**3.** Feedback timing of delayed feedback After a treatment task great benefits in immediate condition A day or several days later—most studies found no significant difference
RQ 2: What theoretical explanations have been applied to explain the results?	No single or main theoretical framework to frame these studies before conduction Six studies did not apply theoretical explanations, while others only reviewed theoretical explanations or discuss theoretical implications in certain sections Theoretical explanations discussed include immediate cognitive comparison, SAT, SCT, the Spacing theory and TAP

This study evaluated the quality of the selected studies based on the quality assessment tool proposed by Kmet et al. (2004). Accordingly, a total of 14 formal criteria were used for assessing quantitative studies, such as sufficient description of questions, evident and appropriate study design, sufficient details of results reported, etc. A total of 10 formal criteria were applied to the assessment of qualitative studies, such as clear context for the study, and clearly described and systematic data collection methods. The inter-rater agreement ranged from 70 to 100%, which is of acceptable quality. If the second researcher disagreed with the first researcher, the two of them discussed the issue with each other until they reached a consensus. No study was excluded based on the quality ratings.

## 4. Limitations

Our systematic review of feedback timing has certain limitations. First, we have only searched and included literature written in English, and possibly overlook some articles with new ideas and methods in other languages. Furthermore, although we have conducted a comparatively thorough search, we may have missed articles that examine the same issue. For the above reasons, we do not claim our review is comprehensive due to the methods applied that could have been more systematic and well-designed. However, we do feel that this review is the first try to focus on both empirical and theoretical issues in feedback timing in SLA.

## 5. Results

### 5.1. Publication year

**Figure 2:**
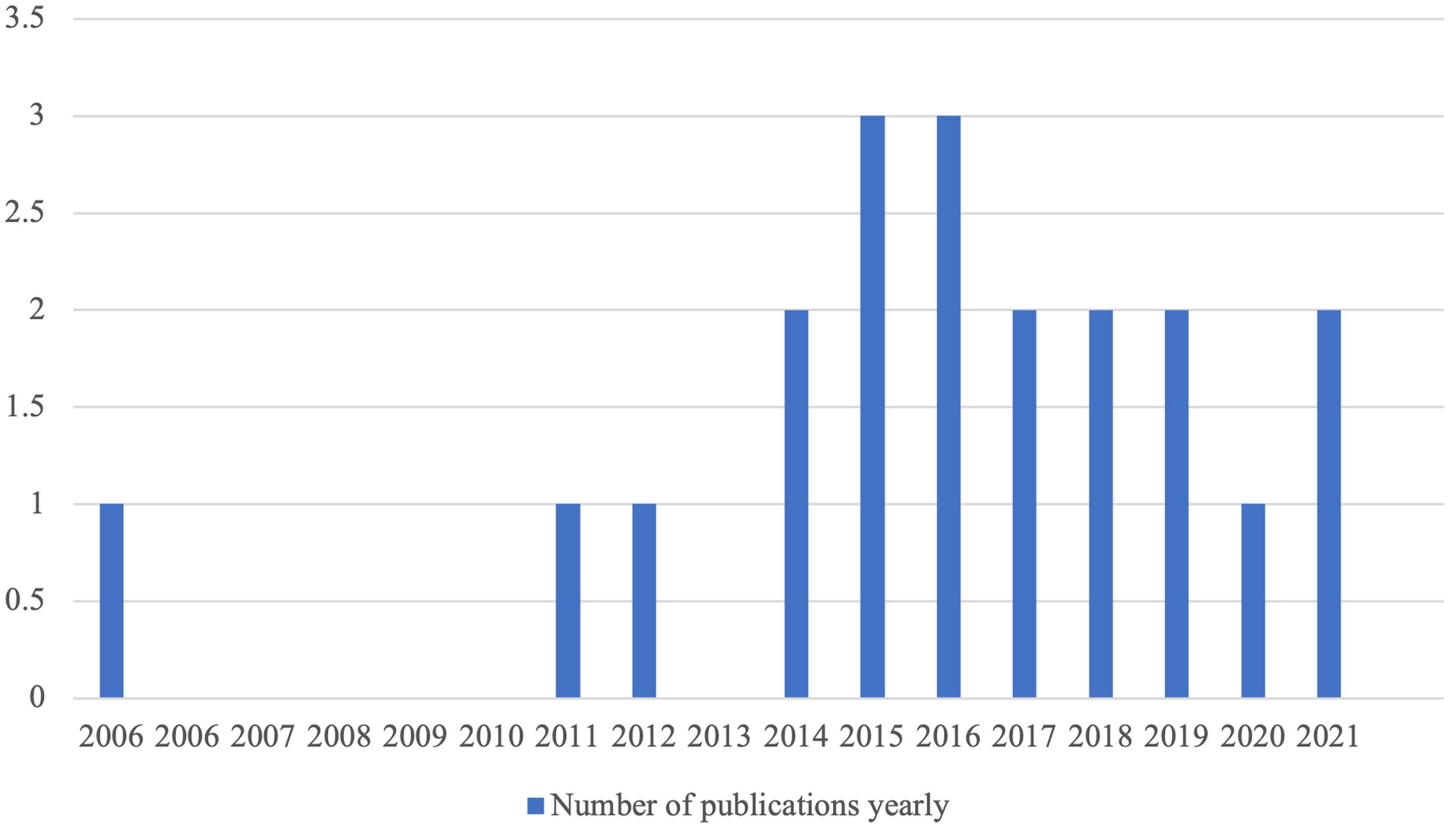
The yearly distribution of the publications included in this systematic review (2006–2021).

Among the 20 studies selected for systematic view, the earliest experimental feedback timing research identified in this study was published in 2006 and the latest in 2021. One article was published in 2006, one article was published in 2011, one article was published in 2012, two articles were published in 2014, three articles were published in 2015, three articles were published in 2016, two articles were published in 2017, two articles were published in 2018, two articles were published in 2019, one article was published in 2020, and two articles were published in 2021. From 2006 to 2011, there were few numbers of research outputs on feedback timing in L2 learning. 2012 saw a gradual increase with respect to the number of scholarly publications in this area, which was likely due to the development of computer-assisted language learning (CALL) and the advantage of providing both immediate feedback and delayed individualized feedback (see Figure 2 for more details).

Considering the importance of the question “when should learner errors be corrected,” and the timing issue was treated addressed in the 1970s (see Hendrickson, 1978, p.389), the total number of 20 was smaller than previously expected.

### 5.2. Descriptive analysis

Before performing the analysis, we conducted a descriptive analysis to summarize the trends and issues of feedback timing in the literature. Of the 20 articles, all but one used quantitative data collection methodologies in their exploration of the impact of feedback timing by applying experimental or quasi-experimental designs. The majority of the presented studies used the pretest-posttest-delayed posttest designs (11 studies), 4 studies used pretest-posttest designs, and 4 studies only applied posttests or post measurements but did not have pre-tests to ensure no significant differences between experimental and control groups. For instance, since Varnosfadrani (2006) compared the effects of immediate and delayed *CF* on learners’ performance of oral English in tailor-mased tests which were created after activities had been finished, the researcher was not able to provide pre-tests. Rahimi and Vahid Dastjerdi (2012) investigated the comparative effectiveness of immediate and delayed error correction methods in developing learners’ complexity, fluency, and accuracy (CAF) in speech. CAF measures as post-tests were developed and showed that delayed feedback has positive effect on fluency and accuracy but not on complexity. The lack of pretest makes it difficult to determine whether the errors are systematic or slips of the tongue. Lavolette et al. (2015) and Shang (2017) investigated English writing by measuring the revised essays and the syntactic complexity of the revised essays, respectively. The above-mentioned studies all considered oral production or written production as a whole and did not use pretests. The lack of pretests of these studies to compare the different feedback timing conditions is problematic because it is impossible to know if the delayed *CF* participants were more capable prior to the *CF* treatment.

There is only one case study (Shintani, 2015) which investigated how two L2 learners responded to synchronous and asynchronous corrective feedback using Google Docs. By analyzing the texts and interview data, Shintani (2015) reported that self-correction was more successful in the SCF condition compared with ACF condition and focusing on meaning and form took place contiguously in the SCF condition while it occurred separately in the ACF condition.

### 5.3. Influential factors

Of the 20 studies, half found immediate feedback outperformed delayed feedback in the post-tests or delayed post-tests, or both. Seven of the comparative analyses found no differences between immediate and delayed *CF*. Only three studies concluded that delayed *CF* was more effective than immediate feedback, but they share limitations due to internal validity issues (Rahimi and Vahid Dastjerdi, 2012; Shang, 2017; Guo, 2018). None of the three studies had control groups that did not receive *CF*. It can be argued that due to the lack of a control group, the improvement that occurs over time cannot be dependably attributed to *CF* treatments. Moreover, there was no pretest in Shang (2017) study. In sum, studies have produced mixed results regarding the relative effectiveness of immediate and delayed *CF* on L2 learning. The following review will mainly focus on the factors influencing the different results.

#### 5.3.1. Communicative modality

Of the presented studies examining the role of feedback in SLA, 40% were conducted in face-to-face communication (eight studies), 30% (six studies) used text-based feedback, 20% (four studies) used computerized feedback provided by computer-assisted language learning (CALL) software, one study used video-based feedback, and one study used text-based feedback as delayed feedback and computerized feedback as immediate feedback.

When studies are conducted in face-to-face oral communication, the findings are mixed. Four studies found immediate feedback condition outperformed the delayed feedback condition (Li et al., 2016a,b; Fu and Li, 2019, 2020), two studies found that delayed feedback was comparatively more effective (Rahimi and Vahid Dastjerdi, 2012; Guo, 2018), and two studies found no difference between the effectiveness of immediate and delayed feedback (Varnosfadrani, 2006; Quinn, 2014).

When studies are conducted in text-based CMC, immediate feedback is argued to be more beneficial to learners than delayed feedback (Shintani, 2015; Shintani and Aubrey, 2016; Arroyo and Yilmaz, 2017, 2018; Yilmaz and Sağdıç, 2019). These studies unanimously agreed that the advantages found for the immediate *CF* on the post-test might be that learners in this timing condition were able to make a cognitive comparison. Only one study found no significant differences between immediate and delayed feedback when learning Spanish vocabulary (Henderson, 2021). Henderson (2021) argued that ‘the salient nature of the modality, type and target of the *CF* may have neutralized a timing effect’ (p. 100).

When studies are conducted in the CALL environment, the results are the same. Feedback timing has been found to have little effect on language learning, such as grammar learning (Henshaw, 2011; Lavolette et al., 2015), vocabulary learning (Nakata, 2014), and writing as a whole (Lavolette et al., 2015). As Lavolette et al. (2015) suggested, in this communicative modality, the different timing did not distinguish the participants’ cognitive resources demands since they were able to go back and see the written text when revising in both feedback timing conditions; in other words, both immediate and delayed conditions involved similar cognitive processes.

As for the only video-based CMC study, Canals et al. (2021) also found no statistically significant differences between the two feedback timing groups. They investigated the relative effectiveness of immediate and delayed *CF* on grammar acquisition by Spanish EFL learners. The delayed feedback provided in this study was contextualized and feedback instances are spaced or naturally spread throughout the interaction. The way of providing delayed feedback with needed information about the target language in context thus improved the effectiveness of delayed *CF*.

Shang (2017) compared synchronous CALL feedback and asynchronous peer feedback. The results showed that asynchronous peer feedback was found more effective than synchronous computerized feedback when comparing students’ syntactic complexity in writing. However, a factor that could account for this finding is the modalities and feedback providers investigated, which means how CALL affected synchronous feedback and how peers in the CMC context affected asynchronous feedback.

#### 5.3.2. The explicitness of the feedback

*CF* treatments were operationalized in different types in the presented studies, such as explicit direct feedback, focused direct feedback, reformulations, partial reformulations, prompts plus recasts, and error repetitions plus recasts, etc. These feedback types can be classified into three categories including explicit, implicit, or hybrid. The majority of the presented studies provided explicit feedback in both feedback conditions (*n* = 10), six studies applied hybrid feedback, and three studies used implicit feedback. One study did not clarify what *CF* types, including the explicitness of the feedback (Rahimi and Vahid Dastjerdi, 2012).

Among the 10 studies providing explicit feedback, most studies found no difference between the effectiveness of immediate and delayed *CF* (*n* = 6). CALL environment tends to provide explicit feedback to language learners (Henshaw, 2011; Nakata, 2014; Lavolette, 2015; Lavolette et al., 2015). Henshaw (2011), for instance, investigated the effects of timing on written *CF* and concluded that written *CF* was effective over time, regardless of its timing. It has been argued that the explicitness of the feedback was especially helpful for learners to confirm or refute interlanguage hypotheses, given that several studies have shown an advantage for explicit feedback, especially in CALL (e.g., Rosa and Leow, 2004). Likewise, those studies that applied more explicit types of feedback in other modalities also tend to find no difference between the two feedback conditions (Varnosfadrani, 2006; Canals et al., 2021). For instance, Canals et al. (2021) investigated the relative effectiveness of immediate and delayed *CF* in video-based CMC and argued the feedback type used in the study, i.e., explicit corrections, might have contributed to no statistical differences between the two feedback timing conditions. Because explicit corrections are relatively direct about the ungrammaticality of the learner’s utterance, they might have promoted the mental comparison between the error and correct alternative more reliably. Shintani (2015) and Shintani and Aubrey (2016), both of which applied focused direct feedback, which is explicit in nature, found SCF was more effective than ACF. This is probably due to the text-based modality they used, which has been discussed in Subsection 5.1.1. As for the two studies that used explicit feedback and found ACF was more effective, proactive feedback of Guo (2018) delayed feedback is likely to be the main cause. Shang (2017) finding is less convincing by comparing feedback provided by different providers, CALL software and peers, in different timing conditions, which can be regarded as a limitation since the difference of feedback providers could act as a confounding variable.

As for studies using implicit feedback, that is, reformulations (Arroyo and Yilmaz, 2017, 2018; Yilmaz and Sağdıç, 2019), it has been consistently found that immediate feedback was comparatively more effective. In oral communication, for instance, Arroyo and Yilmaz (2017, 2018) found that the immediate feedback group outperformed the delayed feedback group in oral production. They argued that the implicit nature of the feedback treatment (declarative reformulations) might lead to the ineffectiveness of the delayed reformulations, thus, they recommended a more explicit or salient treatment when providing delayed feedback.

The six studies providing hybrid *CF* tend to find that immediate feedback was more effective than delayed feedback (Li et al., 2016a,b; Fu and Li, 2019, 2020). While two exceptions are Quinn (2014) and Henderson (2021), both of which found no difference between the two *CF* timing conditions. In Quinn (2021) study, a recast was provided regardless of whether the learner was able to correct themselves, thus making the actual feedback more explicit. As for Henderson (2021), although a hybrid *CF* was claimed to be used, the researcher admitted the salient nature of the *CF* type, because the delayed corrective intent was perceived by learners.

#### 5.3.3. Feedback timing of delayed feedback

The exact time of providing delayed feedback is another important issue to consider when investigating feedback timing. In the presented studies, delayed *CF* could refer to the *CF* that is provided after a treatment task (e.g., Varnosfadrani, 2006; Rahimi and Vahid Dastjerdi, 2012; Quinn, 2014), *CF* provided 24 h later (e.g., Canals et al., 2021), or *CF* provided several days later (e.g., Lavolette et al., 2015).

Henshaw (2011) is the only study that has the length of delay as an independent variable, that is, *CF* immediately after all 40 responses or *CF* 24 h after all responses, but no statistically significant differences were found between different timing conditions.

The majority of the studies used delayed feedback which refers to the *CF* provided after a task or an interview (n = 14). Among those studies providing delayed feedback at the end of the task or after finishing their speech, the results tend to support great benefits for language learning in immediate feedback condition (Shintani, 2015; Shintani and Aubrey, 2016; Li et al., 2016a,b; Arroyo and Yilmaz, 2017, 2018; Yilmaz and Sağdıç, 2019; Fu and Li, 2020). A total of 5 studies found no difference between the effects of the two feedback timing conditions. Varnosfadrani, 2006; Nakata, 2014; Quinn, 2014; Lavolette, 2015; Henderson, 2021). Only one study found error correction provided after participants’ speech had a more positive effect on fluency and accuracy but not on complexity in oral performance than immediate feedback (Rahimi and Vahid Dastjerdi, 2012).

The delayed feedback in three studies was provided a day or several days later. One study (Shang, 2017) using peer feedback required students to provide delayed feedback within 3 days but did not specify how delayed the feedback was. As to studies providing delayed feedback 1 day or several days later, the findings are more consistent since no difference was found between immediate and delayed feedback except in Guo (2018) study. The results provided evidence to the spacing effect hypothesis the delay provides another distributed episode of encoding to solidify the semantic mapping, which in turn, contributes to retention (Butler et al., 2007). In Lavolette et al. (2015) study, the delayed feedback was provided to students as computer-assisted feedback several days (1 to 3 weeks) after their writing essays. The results indicated that the condition – delayed versus immediate – did not affect students’ response rates nor their accuracy on the first drafts. Similarly, in the study by Canals et al. (2021), the delayed feedback group received the feedback 24 h later. In both the grammaticality judgment test and the oral production task, the results showed no differences between the two feedback timing groups.

### 5.4. Theoretical explanations applied

There have been various theoretical explanations for different views about when to provide *CF*. Since feedback timing is an emerging area in SLA research, the present studies share the similarities that there was not a single or main theoretical framework to frame these studies before conduction, in other words, none of them was strongly theoretically oriented.

Six studies (33.3%) did not discuss any theoretical explanations in their studies. Others only reviewed theoretical explanations or discuss theoretical implications in certain sections. Moreover, there are different theoretical explanations for the results about when to provide *CF*. We mainly identified five categories for theoretical explanations that have been applied in the selected studies.

The first theoretical explanation is immediate cognitive comparison (Quinn, 2014; Shintani, 2015; Arroyo and Yilmaz, 2017, 2018; Canals et al., 2021). From this view, a cognitive comparison would take place within a cognitive window. Quinn (2014) argued the reason that there was no difference was that the delayed feedback was not delayed long enough to be different from the immediate *CF* treatment as feedback should be provided within the ‘cognitive window of opportunity for pedagogical intervention’ (p.257), which has been indicated to be less than 1 min. Canals et al. (2021) also argued that no difference was found because of the similar reason.

The second applied theoretical explanation about why immediate *CF* is comparatively more facilitative to L2 development is skill acquisition theory (SAT; Shintani and Aubrey, 2016; Li et al., 2016a,b; Fu and Li, 2019, 2020). For instance, Fu and Li (2020) explained that feedback is more effective before errors are proceduralized through communicative practice. Once proceduralized, linguistic knowledge is programmed into a system and is available for use ‘as a ready-made chuck’ (DeKeyser, 2015, p.95). Therefore, repeated behavioral practice of this kind would be effective.

Sociocultural theory (SCT) was used by Shintani and Aubrey (2016) to explain the comparative advantage of SCF. Suggested by Aljaafreh and Lantolf (1994), the scaffolding in the two treatment tasks reduced gradually in the SCF timing condition. It was only when the researcher identified the learners’ errors did he provide interactive SCF in the ongoing writing process. Such a gradual shift within the two writing tasks did not occur in the ACF group, indicating that SCF provided more optimal scaffolding for learners than ACF did.

As to those explanations that support delayed feedback, the most applied one is the Spacing theory from cognitive psychology (Nakata, 2014; Li et al., 2016a,b; Guo, 2018). Although Nakata (2014) found feedback timing may have little effect on learning, the theoretical explanation used supports delayed feedback. Nakata (2014) argued that larger spacing would generally lead to better long-term retention compared with short spacing, which can be called distributed practice. However, in this study the lack of a significant feedback timing effect was suggested because the beneficial effects of delaying feedback which involves larger spacing, and less interference might have been offset by the risk of not correcting an error immediately. As to Guo (2018) study, which found learners in the delayed feedback condition produced better final test performance than those in immediate feedback condition, it was argued that the results provided evidence to the spacing effect hypothesis the delay provides another distributed episode of encoding to solidify the semantic mapping, which in turn, contributes to retention (Butler et al., 2007).

The last theoretical framework applied in these studies to explain L2 knowledge development from either immediate or delayed feedback is TAP (Arroyo and Yilmaz, 2017; Henderson, 2021). According to TAP, providing grammar instruction which is integrated into communicative activity may lead to an increase in implicit knowledge while providing isolated grammar instruction may lead to an increase in explicit knowledge. Because immediate *CF* is provided during a communicative activity, it is arguably integrated grammar instruction. If *CF* were to be delayed until after a communicative activity, arguably, it could be considered isolated grammar instruction. In this interpretation, immediate and delayed *CF* is likely to result in greater development of implicit and explicit knowledge, respectively. Arroyo and Yilmaz (2017) found that immediate *CF* was significantly more facilitative in the oral production test (OPT), which was a meaning-based and contextualized task. In this way, the results of the OPT that immediate feedback was more facilitative are consistent with TAP. The lack of outperformance of the delayed *CF* in grammaticality judgment test (GJT), which was decontextualized, was possibly due to implicit nature of the feedback.

## 6. Discussion

The studies we have reviewed pointed out that there is not a simple model of perfect *CF* timing pedagogy. Overall, this literature review showed that immediate feedback was more effective or equally effective compared with delayed feedback. The different findings could result from the different communicative modalities used, different degrees of explicitness of the feedback and the exact time of providing delayed feedback. Having described the different designs adopted in these empirical studies, we now turn to a discussion of the areas this review has highlighted for improving future study quality.

An interesting finding that emerges from this literature review is the lack of attention to *CF* in CMC modalities other than text-based interaction, which tend to support the beneficial role of immediate feedback due to the salience of the modality that could neutralize the timing effect. As to the only study conducted in a video-based CMC context, the asynchronous feedback was provided 24 h later by means of an edited video recording of the interaction (Canals et al., 2021). In other words, the delayed feedback processing was contextualized and thus learners were able to link the errors that were represented to them. The limitation is the lack of ecological validity as it could cost a great deal of time to provide delayed feedback. Considering the current COVID-19 pandemic, synchronous online teaching using tools, such as Zoom and Tencent Meeting, has become more widespread. Communication in SCMC can be text-based, audio-based, video-based, or multimodal (a combination of text, audio or video). Moreover, with the development of smartphone technology, mobile-assisted language learning has received attention in language learning as it is largely free of time and location constrains and thus, convenient for language learning. In particular, the advance of mobile technologies with social communication features, such as the ability to review content (in text or audio) and then provide comments accordingly, makes it possible for teachers to provide feedback *via* test, voice, and video images. Nevertheless, little research has been conducted to investigate the characteristics of feedback using social communication apps, let alone the ideal time to provide *CF*. Since communication modality can influence the amount and nature of interactional features that occur naturally in communication, further research could be conducted in different modalities.

Our review has shown that the issue of the construct of ‘delayed feedback’ needs to be clarified and unified in future studies. Even though all the presented studies on feedback timing compared the immediate and delayed feedback, little discussion has been made about the definition of delayed *CF* and the boundary between immediate and delayed *CF*. The original definition of immediate correction as correction that interrupts utterances, and delayed correction as correction that allows learners to finish their utterances (Chaudron, 1977; Long, 1977). Li et al., (2016a,b) argued that the timing of *CF* can be classified at least in two ways: immediate versus delayed and online versus offline. The former refers to whether errors should be corrected immediately after learners receive instruction on a certain linguistic structure or sometime later after the instruction. The latter refers to whether errors should be corrected during a communicative task or after finishing it. Future research is needed to clarify and unify the term ‘feedback timing’. Apart from online and offline (context), immediate and delayed (schedule), the *CF* timing constructs in those studies must be well-defined. One research question that specifically needs investigation is whether delayed *CF* is a monolithic construct. Future researchers could also further investigate whether a difference in the length of delay makes the findings different in other modalities and learning other target structures. In other words, it should be considered whether there are empirical differences in the effects of end-of-task, end-of-class, subsequent-day delayed *CF*, and several-days delayed *CF*.

The effects of feedback timing may be mediated by the nature of linguistic targets, therefore, there should be more linguistic targets, which refer to what to correct in feedback, considered in future studies. In the existing studies, feedback is provided on a very restricted range of linguistic errors, which does not resemble what really happens in real language classrooms. The research has mainly been conducted in grammatical category. However, grammar learning in these studies, such as hypothetical conditional structure, is complex but also rule based. Shintani et al. (2014) found that, whereas traditional direct written *CF* had a positive effect on the accuracy of the hypothetical conditional, it had no effect on the indefinite article, a particularly nonsalient feature. One area for future investigation would be whether immediate and delayed *CF* is effective in helping learners acquire such non-salient features (Shintani and Aubrey, 2016). Other linguistic focuses, such as lexical, phonological, and pragmatic, have seldom been investigated. Kamiya and Nakata (2021) mentioned that an interesting area of research would be to examine the effects of timing of oral *CF* on vocabulary learning. In practice, oral *CF* can be provided immediately after the learner’s erroneous utterance (immediate OCF) or after a delay (delayed OCF). Although cognitive psychology literature on the distributed practice effect predicts the advantage of delayed OCF over immediate OCF, existing studies on grammar learning have provided little evidence for the benefits of delaying OCF (Quinn and Nakata, 2017). However, it is possible that vocabulary acquisition benefits more from delayed OCF than grammar acquisition. This is because research has shown that the timing of instruction, not OCF, has larger effects on vocabulary learning (Nakata, 2015; Nakata and Webb, 2016) than grammar learning (e.g., Suzuki and DeKeyser, 2017). In future research, it may be useful to examine whether delaying OCF facilitates vocabulary learning compared with immediate feedback.

As to the methodological issues, one positive trend in the studies we reviewed was the widespread use of the control group in pretest-posttest-delayed post-test design. Without control groups, an improvement over time cannot be convincingly attributed to the *CF* treatments but improvement over time, which is a very important internal validity issue. However, feedback conditions in some studies differ not only in terms of timing but also in terms of other factors that could have been confounded with timing, such as feedback types. For instance, the *CF* used for immediate *CF* differs in type from the *CF* used for delayed *CF*, which makes it difficult to claim that differences in outcomes are the result of *CF* timing differences. Future research should employ a rigorous methodology that avoids confounding *CF* type and timing and includes no-*CF* control groups and post-test. Secondly, more long-term *CF* timing studies should also be conducted to determine whether the effects found in one-off studies are maintained over longer time periods. Thirdly, future research is needed to investigate whether the results would be the same for advanced learners and beginners or not. Additionally, other research can be undertaken to compare the effects on learners of different age groups. As Nakata suggested in his study, it can be suggested that the findings would be of difference in the age of participants (grade school vs. college students). In addition, it would be advisable for future studies to include introspective methods (e.g., stimulated recall and think aloud) to provide further insight into how learners process immediate and delayed *CF* in different contexts and what additional factors might contribute to such processing (Arroyo and Yilmaz, 2017, 2018; Henderson, 2021). Last but not least, more replication studies should be done to advance scientific knowledge by establishing the generalizability of the research findings (Marsden et al., 2016). Despite the benefits of replication, little replication research has been conducted in the area of *CF* in recent years. To increase the certainty in the results reported, the full set of data and research instruments should be publicly available online to researchers. Future *CF* timing researchers should follow Arroyo and Yilmaz (2018) in making their materials available to assess the generalizability of the present findings.

As to the theoretical explanations, it can be concluded that various theoretical explanations exist for why immediate or delayed *CF* may be effective. However, empirical studies under a solid theoretical framework which is important to direct researchers through common challenges in the development, collection, and analysis of research (Ravitch and Riggan, 2017). Arroyo and Yilmaz (2018) also argued that the L2 acquisition literature is rich with regard to theoretical perspectives that can be used to justify the provision of immediate or delayed feedback, but the focus-on-form (Long, 1991) perspective is probably the only theoretical framework that has made an explicit claim about the relationship between feedback timing and the effectiveness of feedback, which suggests the lack of theoretical framework applied in previous studies. Apart from the theories discussed in the studies, other theoretical frameworks could be applied to explore the timing of providing *CF*. For instance, according to the desirable difficulty framework (Schmidt and Bjork, 1992), delayed feedback (summarized) was better than immediate (continuous) feedback during practice as the former tends to slow down the initial learning and result in better retention in the long run. Suzuki et al. (2019) pointed that feedback timing was an emerging area and could be conducted in the scope of L2 practice and desirable difficulty framework.

## 7. Conclusion

Feedback timing is an issue of great importance to investigate. The studies we have reviewed pointed out that there is not a simple model of perfect *CF* timing pedagogy. Overall, this literature review showed that immediate feedback was more effective or equally effective compared with delayed feedback. This study adds to our knowledge that the differences in terms of the comparative effectiveness of immediate and delayed feedback could result from the different communicative modalities used, different degrees of explicitness of the feedback and the exact time of providing delayed feedback.

## Data availability statement

The original contributions presented in the study are included in the article/Supplementary material, further inquiries can be directed to the corresponding author.

## Author contributions

MX: conceptualization of the review, literature search, writing of the original draft, revision, and editing of the manuscript. SZ: revision and editing of the manuscript. All authors contributed to the article and approved the submitted version.

## Funding

This study was supported by Minzu University of China in 2021 (No. 2021QNPY69) and Shenzhen Federation of Humanities and Social Sciences (grant number SZ2020B031).

## Conflict of interest

The authors declare that the research was conducted in the absence of any commercial or financial relationships that could be construed as a potential conflict of interest.

## Publisher’s note

All claims expressed in this article are solely those of the authors and do not necessarily represent those of their affiliated organizations, or those of the publisher, the editors and the reviewers. Any product that may be evaluated in this article, or claim that may be made by its manufacturer, is not guaranteed or endorsed by the publisher.
